# Special Issue: Mechanical Properties in Progressive Mechanically Processed Metallic Materials

**DOI:** 10.3390/ma13204668

**Published:** 2020-10-20

**Authors:** Radim Kocich, Lenka Kunčická

**Affiliations:** 1 Faculty of Materials Science and Technology, VŠB-Technical University of Ostrava, 70833 Ostrava 8, Czech Republic; 2 Institute of Physics of Materials, Czech Academy of Sciences, 61662 Brno, Czech Republic

**Keywords:** mechanical properties, functional properties, metallic systems, mechanical processing, structural phenomena

## Abstract

The research and development of modern metallic materials imparts not only the introduction of innovative alloys and compounds, but also the increasing lifetime of existing materials via optimized deformation processing. Among the essential features of progressive metallic materials used for modern applications are enhanced mechanical properties, but also other high-level functional characteristics, such as thermal–physical parameters, corrosion rate, and electric resistance. The properties of materials and alloys ensue from their structures, which can primarily be affected by the preparation/production process. The Special Issue “Mechanical Properties in Progressive Mechanically Processed Metallic Materials” was established to present recent developments and innovations particularly in the engineering field. The Special Issue comprises papers dealing with modern materials, such as metallic composites and pseudoalloys, as well as developments in various processing technologies.

The demands on innovative materials given by the ever-increasing requirements of contemporary industry impart the usage of high-performance engineering materials, among the most innovative ones are multicomponent materials, such as gradient structures and composites, which are able to satisfy top-level individual requirements through the possibility to benefit from the advantages of all their components. The Special Issue contains two papers dealing with the preparation of modern aluminum/copper electro-conductive clad composites. One of the papers primarily focuses on numerical prediction and experimental evaluation of the deformation behavior of both the component metals during processing [1], whereas the other is a thorough study of the internal structure, texture in particular, of the reinforcing wires [2]. The composites are prepared via the innovative method of severe plastic deformation (SPD) of twist channel angular pressing (TCAP), the thorough review of which is performed in another paper [3].

Among the possible ways of how to effectively increase the utility properties of metallic materials is to decrease their grain size. In addition to using the SPD methods based on imposing severe shear strain resulting in grain refinement introducing enhancement of numerous properties, powder metallurgy can be applied to provide powders featuring fine grain sizes at the very beginning of the production process. The paper by K. Dvořák et al. [4] presents the possibility of using modern disintegrators to prepare fine powder particles. Several papers published within the Special Issue then deal with processing of metallic powders—assessing and optimizing the processing conditions and evaluating various phenomena of the final products. The paper by J. Málek et al. [5] deals with the effects of the selected processing route on the properties of a biocompatible HfNbTaTiZr high entropy alloy (HEA), for the specific advantageous properties of HEAs have been within the focus of researchers for the last few decades, whereas other researchers focused on a WNiCo tungsten heavy alloy, from evaluating and optimizing the sintering conditions for the initial powders [6], through numerical simulation of deformation behavior during plastic deformation processing [7], to thorough investigation of structural phenomena, such as microstrains and residual stress [8].

The introduction of thermomechanical treatment represented a breakthrough in grain refinement, consequently leading to significant improvement of the mechanical properties of metallic materials. Contrary to conventional production technologies, the main advantage of such treatment is the possibility to precisely control structural phenomena, including grain size, substructure development, texture, and volumes and types of grains boundaries, all of which affect the final mechanical and utility properties. The strengthening mechanisms in modern high-temperature resistant alloyed steels after heat treatment were studied by L. Zhao et al. [9], while A. Olina et al. [10] investigated the occurrence of retained austenite in a fine-grained spring steel processed via thermomechanical rolling. J. Fumfera et al. [11] then numerically and experimentally evaluated the cyclic hardening behavior in dependence with the strain range for a 08Ch18N10T austenitic stainless steel.

Last but not least, the semi-products fabricated from modern materials have to be finished to reach their final shapes and forms, which can be a challenge considering their enhanced properties and unique structures. Nevertheless, there are cutting and shaping methods that can be advantageously used for these purposes. The well-known machining process is suitable for the finishing of numerous products; however, the process introduces micro-deformations to the materials being processed and this factor should be considered as it can alter the lifetime and properties of the final product [12]. Additionally, for this reason, the abrasive water jet cutting technology can be considered as very favorable for cutting and shaping modern materials [13,14].
